# Hot Air Currents

**Published:** 1914-05

**Authors:** 


Hot Air Currents. Henry (sic) Rozies of Ste-Maxime-sur-Mer, *Le Progres Med.,* Nov. 27, 1913, explains that hot air treatment by the confined air bath is limited mainly to osteo-articular affections, the hot air blast may be used on ulcers, atonic wounds, burns, in neuralgias and neurities, even against the lesion of diphtheria. Analgesic, antiseptic, alterative, cicatrisant and other actions are obtained by temperatures of 100-200 (C.), while temperatures of 200-300 may be used as caustics. The air may be heated by hand-compressed bulbs, so as to pass it through the flame of a Bunnsen burner or the compressed air tanks of the Compagnie Poop may be used in the same way. Bellows worked by the foot may also be employed. Various electric apparatuses are mentioned, in which the current operates a fan and also a resistance coil to warm the air.

